# Portable Microfluidic Viscometer for Formulation Development and in Situ Quality Control of Protein and Antibody Solutions

**DOI:** 10.1021/acs.analchem.4c02099

**Published:** 2024-08-02

**Authors:** Philippe S. Lenzen, Fabian Dingfelder, Marius Müller, Paolo Arosio

**Affiliations:** † ETH Zürich, Department of Chemistry and Applied Biosciences, Institute for Chemical and Bioengineering, 8093 Zürich, Switzerland; ‡ Janssen R&D, BTDS Analytical Development, 8200 Schaffhausen, Switzerland

## Abstract

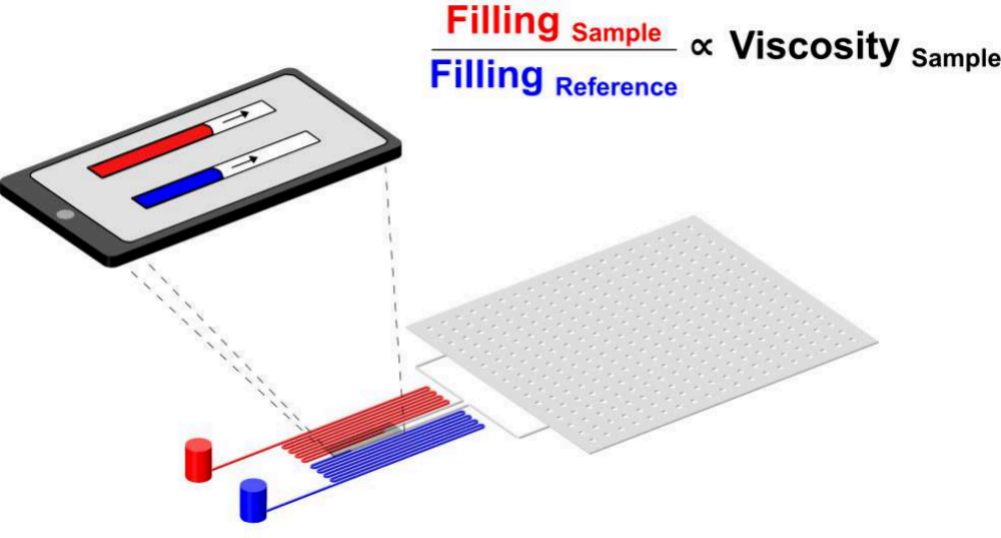

Viscosity of protein solutions is a critical product quality attribute for protein therapeutics such as monoclonal antibodies. Here we introduce a portable single-use analytical chip-based viscometer for determining the viscosity of protein solutions using low sample volumes of 10 μL. Through the combined use of a microfluidic viscometer, a smartphone camera for image capture, and an automated data processing algorithm for the calculation of the viscosity of fluids, we enable measurement of viscosity of multiple samples in parallel. We first validate the viscometer using glycerol–water mixtures and subsequently demonstrate the ability to perform rapid characterization of viscosity in four different monoclonal antibody formulations in a broad concentration (1 to 320 mg/mL) and viscosity (1 to 600 cP) range, showing excellent agreement with values obtained by a conventional cone–plate rheometer. Not only does the platform offer benefits of viscosity measurements using minimal sample volumes, but enables higher throughput compared to gold-standard methodologies owing to multiplexing of the measurement and single-use characteristics of the viscometer, thus showing great promise in developability studies. Additionally, as our platform has the capability of performing viscosity measurements at the point of sample collection, it offers the opportunity to employ viscosity measurement as an in situ quality control of therapeutic proteins and antibodies.

## Introduction

Numerous therapeutic proteins such as monoclonal antibodies (mAbs) are formulated as liquid solutions for subcutaneous self-administration. Given the low-volume requirements for subcutaneous injections, an appropriate dosing requires these proteins to be formulated at high concentration (≥150 mg/mL).^1,2^ At these concentrations, molecular surface properties such as charge distribution and hydrophobicity mediate weak multivalent protein–protein interactions that can induce reversible self-association of the drug products.^3−10^ The result is the formation of transient and highly dynamic protein clusters and networks that can resist fluid deformation and macroscopically manifest as either opalescence or high viscosity.^11−17^ A high tendency for nonspecific interactions can have deleterious effects on the entire chain of the drug products and high viscosity can induce pain during injection. As such, special precautions are implemented during the formulation of these drug products to ensure protein stability while simultaneously optimizing the viscosity of the formulation.

For this purpose, there is a desire for analytical tools capable of measuring the viscosity of high concentration antibody formulations with good throughput for screening studies and with a high dynamic range suitable for a broad range of products. Given the high protein concentration, these analytical methods must be compatible with low sample volumes to offer a cost-effective viscosity measurement. In this context, microfluidic approaches are well suited given their low volume requirements, their exquisite flow control, as well as the cost benefit they offer. Several microfluidic devices have been proposed to characterize the biophysical properties of protein solutions, including for the measurement of viscosity of therapeutic proteins.^18−24^

The most notable example exploits the Poiseulle flow at the micron-scale to measure the pressure drop across a channel while ensuring a constant flow rate through the use of syringe pumps.^25^ Alternative methods measure viscosity by coflowing the solution of interest and a reference fluid in a microfluidic channel and measuring the position of the interface between the two solutions.^26,27^ One interesting approach exploits the gas solubility of the polymeric matrix of the device to induce fluid motion following degassing of the matrix. In this strategy, viscosity is evaluated by comparing the filling rate of the solution of interest with a reference liquid of known viscosity.^28^

While these methods enable a reliable measurement of viscosity, limitations exist either in terms of sample volume requirement, or experimental throughput. In addition, viscosity measurements remain limited to settings where these analytical tools and the various peripherals needed are available.

In this work, we develop a microfluidic platform for determining the viscosity of multiple samples simultaneously through the combined use of a microfluidic viscometer, a portable image acquisition device, and an automated data processing algorithm for the calculation of fluid viscosity. This analytical method enables the simultaneous measurement of 5 solutions using as low as 10 μL of high concentration protein formulations, therefore offering the throughput and minimal sample consumption required for development studies. In addition to multiplexing, this single-use viscometer also alleviates the current drawbacks presented by the extensive cleaning protocols in place today. The portable chip-based viscometer enables the measurement with simple user operation at the point of sample collection, which opens the attractive opportunity to use of the device for in situ quality control of drug products.

## Experimental Section

### Materials

The four IgG1 monoclonal antibodies evaluated in this study were provided by Janssen R&D (Schaffhausen, Switzerland) and labeled mAb1 to mAb4. The monoclonal antibodies were provided at various concentrations ranging from 1 mg/mL to 320 mg/mL depending on the specific antibody. All dilutions were performed in the appropriate formulation buffer.

### Microfluidic Device Design and Fabrication

Silicon wafer templates were fabricated using standard SU-8 photolithography following the manufacturer’s protocol (SU-8 3025, Kayaku Advanced Materials, USA) and served as negative molds for replication of microfluidic devices using soft-lithography of polydimethylsiloxane (PDMS) (Sylgard 184 kit, Dow Corning, USA) by curing at 100 °C for 30 min. The PDMS replicate containing the microfluidic channels was first cut, punched using 3 mm biopsy punch (Kai Medical, Japan) and subsequently bonded to a glass slide (Corning, USA) following plasma activation (Zepto plasma cleaner, Diener Electronics, Germany). The fabricated microfluidic viscometers were subsequently packaged in a vacuum using a commercial vacuum instrument (Solis Vac Premium, Switzerland). To ensure hydrophobicity of the PDMS surface, the fabricated and packaged viscometers were stored for a minimum of 24 h before use and remain usable for weeks following packaging.

### Measurement of Liquid Viscosity

Prior to a measurement of viscosity, the antibody formulations were allowed to equilibrate at room temperature for 30 min. Following this equilibration period, the vacuum-packaging was opened, and the microfluidic device was placed on an illuminated surface (Kaiser Slimilte, Germany). Subsequently, 10 μL of each solution of interest was pipetted in a corresponding inlet. For measurement of the filling rate in the measurement channels, two methods were employed: a smartphone camera (Pixel 6a, Google, USA) or a stereomicroscope (2000-C, Zeiss, Germany) equipped with a reflex camera (EOS 550D, Canon, Japan). Images were captured at a frame rate ranging from 24 to 50 frames per second.

As a standard reference fluid for the calculation of viscosity, ultrapure water was used with a known dynamic viscosity of  = 0.9544 cP at 22 °C and 1 atm. For the measurements of samples with a viscosity exceeding 300 cP, a glycerol (ABCR, Germany) solution was used as the reference liquid. We estimated that the microfluidic viscometer provides reliable viscosity ratio between the measured sample and the reference fluid below or equal a value of 50. All measurements were performed at room temperature between 21 and 23 °C.

### Analysis of Liquid Filling Rates

The analysis of the liquid filling rates was performed automatically through an automated python script. The image sequence was initially imported, a Gaussian blur filter was applied to mitigate noise and enhance contrast, and a rolling ball background subtraction algorithm was implemented. Subsequently, the image series was aligned to a known mask of the device using an image alignment algorithm. Following which, the position of the fluid front in the measurement channels was obtained for each image, and the displacement of the fluid front was calculated for the entirety of the image series. The filling rate was then calculated as the displacement of fluid front over time and subsequently averaged over multiple frames to ensure accuracy and reproducibility of the measurement.

### Rotational Cone and Plate Rheometer Measurements

Rotational cone and plate measurements of viscosity were performed on a Haake Rheostress 600 (Thermo Fisher Scientific, USA). The rheometer was set up using a rotation time of 300 s, a 60 mm cone, an angle of 1°, and a gap distance of 0.052 mm. For each measurement, 500 μL of antibody solution was used and all the measurements were performed at shear rates of 200 s^–1^. The viscosity values reported were derived from the y-intercept of a linear regression of viscosities plotted against rotation time. All measurements were performed at 20 °C.

## Results and Discussion

### Microfluidic Viscometer for Simultaneous Measurement of Multiple Liquids

**Figure 1:**
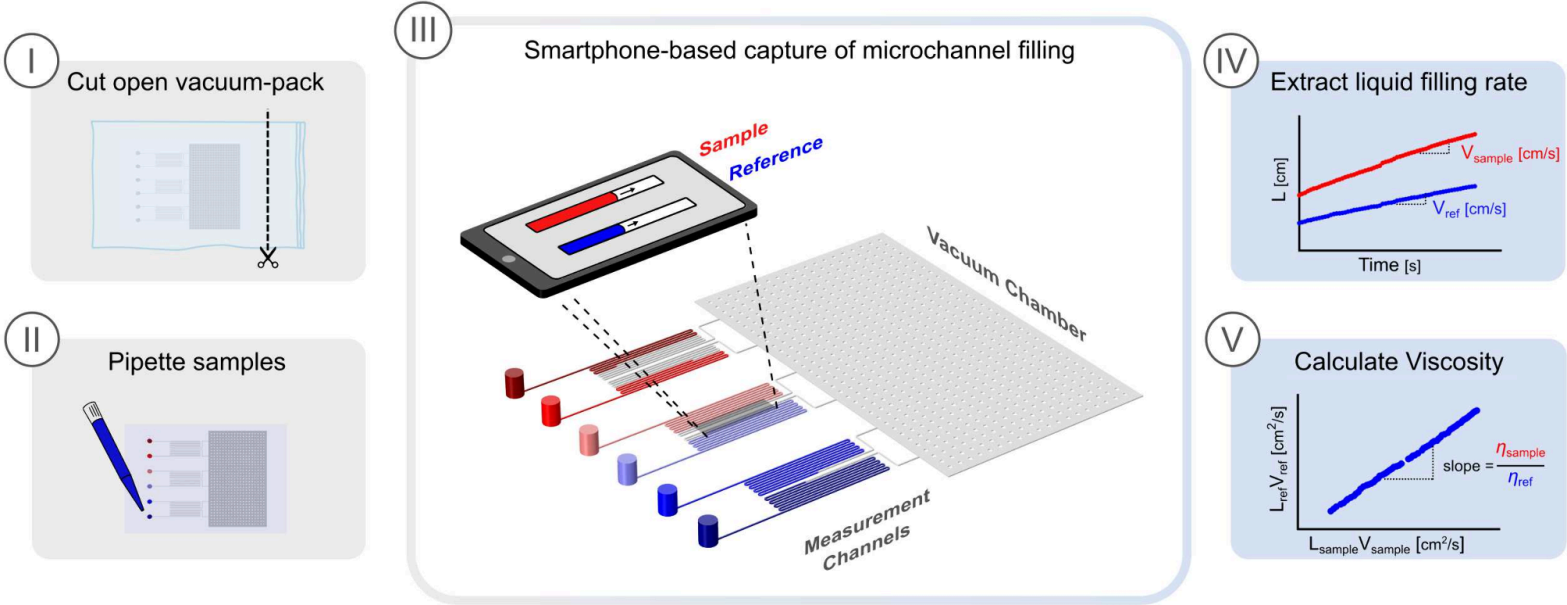
Schematic illustration of the user operation and principle of the portable microfluidic viscometer. (I) Cut open the vacuum-packaging. (II) Pipette the samples in the microfluidic viscometer. (III) Image the filling of the channels using a smartphone camera. (IV) Plotting the displacement of the fluid front over time for both sample and reference and extract each filling rate. (V) Calculation of viscosity

**Figure 2:**
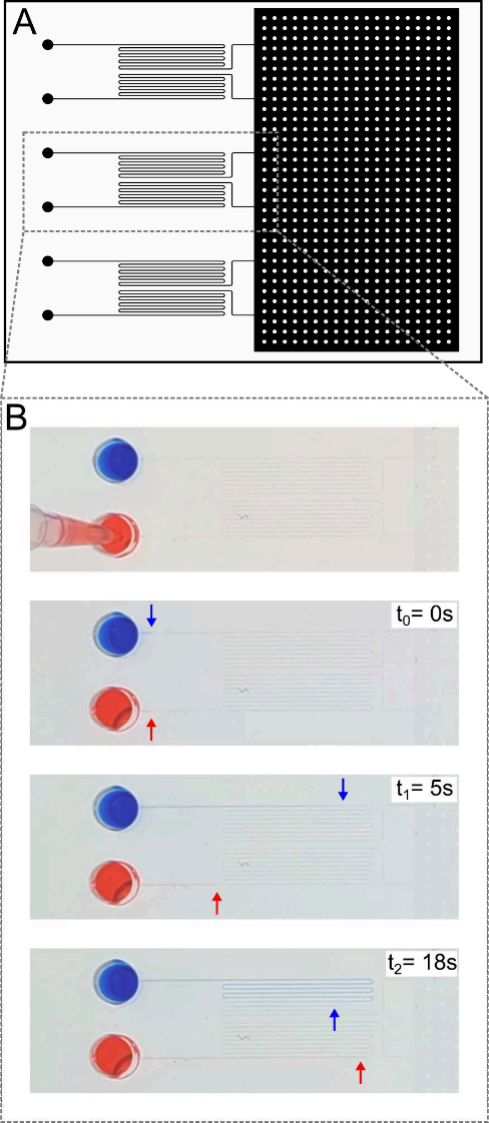
(A) Design of the microfluidic viscometer. (B) Time-lapse images of two solutions, one with low viscosity (blue) and another with high viscosity (red). Both solutions were dyed to facilitate visualization.

A schematic illustration of the user operation and principle of the viscometer is shown in Figure 1. The microfluidic viscometer is composed of six identical microchannels (10 cm length and 100 μm width). Each channel is open at one end to allow for pipetting the samples in the device, and all channels are connected at the other end of the device to a single vacuum chamber (see Figure 2A).

The filling of the channels is induced by exploiting the gas solubility of the polydimetylsiloxane (PDMS) microfluidic device. Specifically, prior to a measurement, the device is placed in a vacuum to remove soluble gas molecules from the PDMS walls of the channel. Once the device is brought back to ambient pressure, gas molecules diffuse back into the PDMS matrix, which generates the pressure required to drive the flow of liquid from the inlets into the measurement channels, a process governed by the Hagen–Poiseuille law:
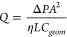
1where *Q* defines the volumetric flow rate, η the dynamic viscosity, Δ*P* the pressure difference across the channel, *A* the channel cross-sectional area, *L* the length traveled by the fluid, and *C*_*geom*_ a dimensionless geometrical correction factor related to the channel geometry.^29^ Due to the inherent variability of the pressure governing the fluid filling in the microchannels, the measurement of fluid viscosity cannot be accomplished only by analyzing the sample fluid of interest. To circumvent this issue, an additional reference solution is introduced in the device simultaneously with the samples of interest. Representative images showing liquid filling in the measurement channels are shown in Figure 2B, in which a low viscosity (blue) and a high viscosity (red) solutions flow in their respective microchannels.

Owing to the geometrical design of the microfabricated viscometer, the pressure driving the filling of liquids in the measurement channels is equally distributed in all channels, and ensures that the pressure drop across the measurement channel of a reference solution is equal to the pressure drop across the samples.

By measuring the volumetric flow rates Q of both the sample and the reference solution of known viscosity (η_*ref*_) in two identical microchannels, the unknown viscosity (η_*sample*_) is obtained by the following relationship, which is derived by equalizing the pressure drop across both the sample and reference measurement channels:
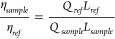
2

The captured images are first analyzed to extract the microchannel filling distance (*L*), from which the filling velocity (*V*) is then calculated at each time interval, where  and *A* is the channel cross-sectional area (Figure 1-IV). By multiplying *L* and *V*, and plotting the resulting data for the sample and reference solutions (Figure 1-V), the viscosity of the sample solution is obtained by calculating the slope of the fitted values according to eq 2.

To increase the experimental throughput, the microfluidic viscometer was developed with six identical measurement channels connected to a single vacuum chamber. Through this parallelization, five measurements can be performed simultaneously in less than 5 min (not including data analysis).

### Portable Viscometer for Measurement at the Point of Sample Collection

To enhance the accessibility and flexibility of the microfluidic viscometer, a number of features were implemented with the aim of enabling portable viscosity measurements. These enhancements allow for a measurement to be conducted with minimal peripheral equipment for liquid actuation and analytical readout, thereby eliminating the need for sample transport and enabling a viscosity measurement to be conducted directly at the collection site.

First, the microfluidic devices manufactured were stored in vacuum packaging until use. This ensures that the pressure required to drive the flow of liquids is only generated once the device packaging is opened and the microfluidic viscometer is brought back to ambient pressure (Figure 1). The vacuum packaging maintains a consistent low pressure for weeks after sealing, ensuring a steady flow generation. Additionally, it enables safe transport and storage of microfluidic devices without the risk of contamination. This combination of portable flow generation and pipette-based operation greatly improves the ease of use and simplicity of operation of the microfluidic viscometer.

Second, as to provide a comprehensive capability of measurement at the point of sample collection, the capture of the displacement of the fluid front was implemented using a smartphone camera. As a result, the measurement can be performed without any benchtop instrumentation, thereby facilitating its implementation at the collection site.

Third, an algorithm was developed to automate the measurement of viscosity. The system automates the image and data analysis process, extracting the filling of fluids in the microchannels and automatically calculating the viscosity based on the input of the reference fluid viscosity. Currently, the angle of the smartphone camera affects the analysis of liquid filling. However, the implementation of a homography transformation in the algorithm would ensure consistent analytical performance when using hand-held imaging.

Combined, these advancements enable a comprehensive and portable viscosity measurement at the sample collection point with minimal equipment.

### Validation of the Microfluidic Viscometer Using Glycerol–Water Mixtures

**Figure 3:**
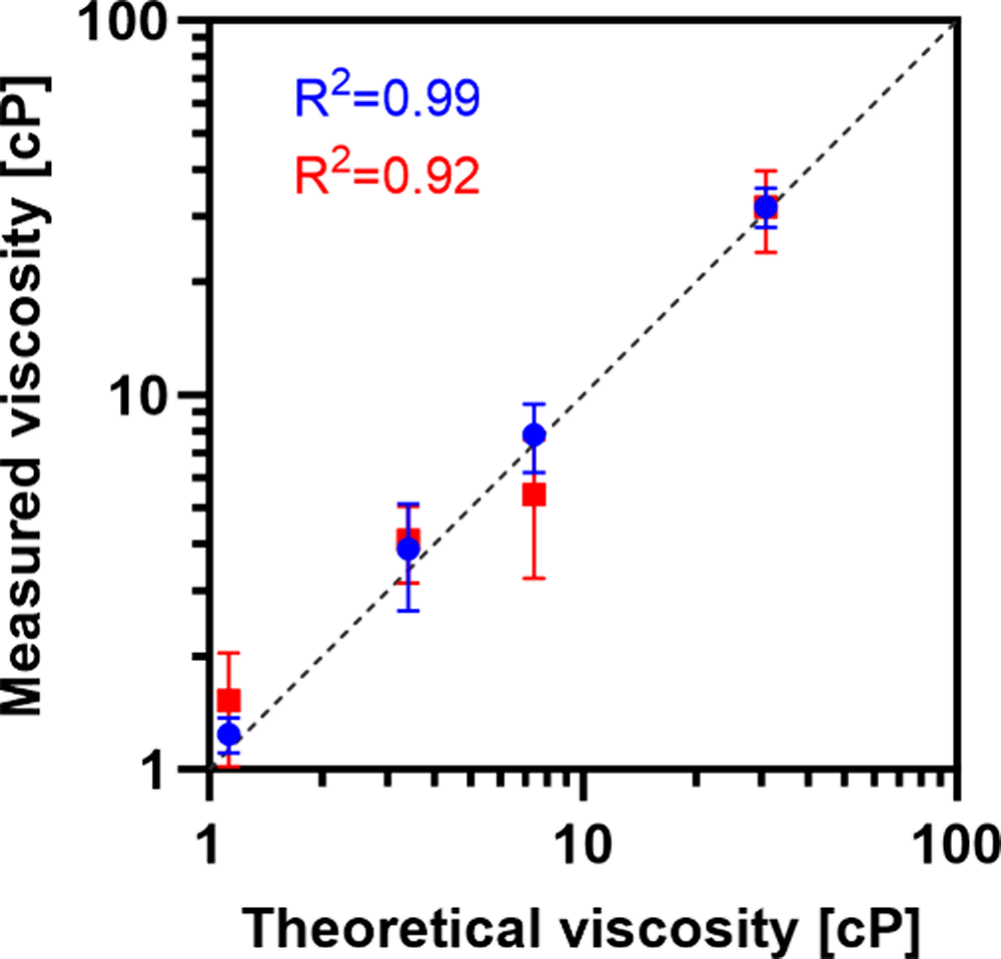
Validation of the microfluidic viscometer using four different glycerol–water mixtures with glycerol concentrations of 10, 40, 55, and 75 wt %. Images were collected using either a stereomicroscope (blue circles) or a smartphone camera (red squares). In both cases, we observed excellent agreement with theoretical values (correlation coefficients of *R*^2^ = 0.99, and *R*^2^ = 0.92, respectively). The values correspond to means and standard deviations of triplicate experiments.

The microfluidic viscometer developed in this study was first validated by measuring the viscosity of different glycerol–water mixtures, as shown in Figure 3.

Four different glycerol–water mixtures of 10, 40, 55, and 75 wt % where measured in triplicate experiments. The results showed an excellent correlation between the viscosity values measured using the microfluidic viscometer and the theoretical viscosity values,^30^ with a correlation coefficient *R*^2^ = 0.99 using a stereomicroscope for the measurement. Similarly, a high correlation of *R*^2^ = 0.92 was observed when using a smartphone camera for liquid filling measurement.

### Viscosity of High Concentration Antibody Formulations

We next applied the microfluidic platform to measure the viscosity of four different monoclonal antibodies at high concentration, each in their specific formulation buffer.

**Figure 4:**
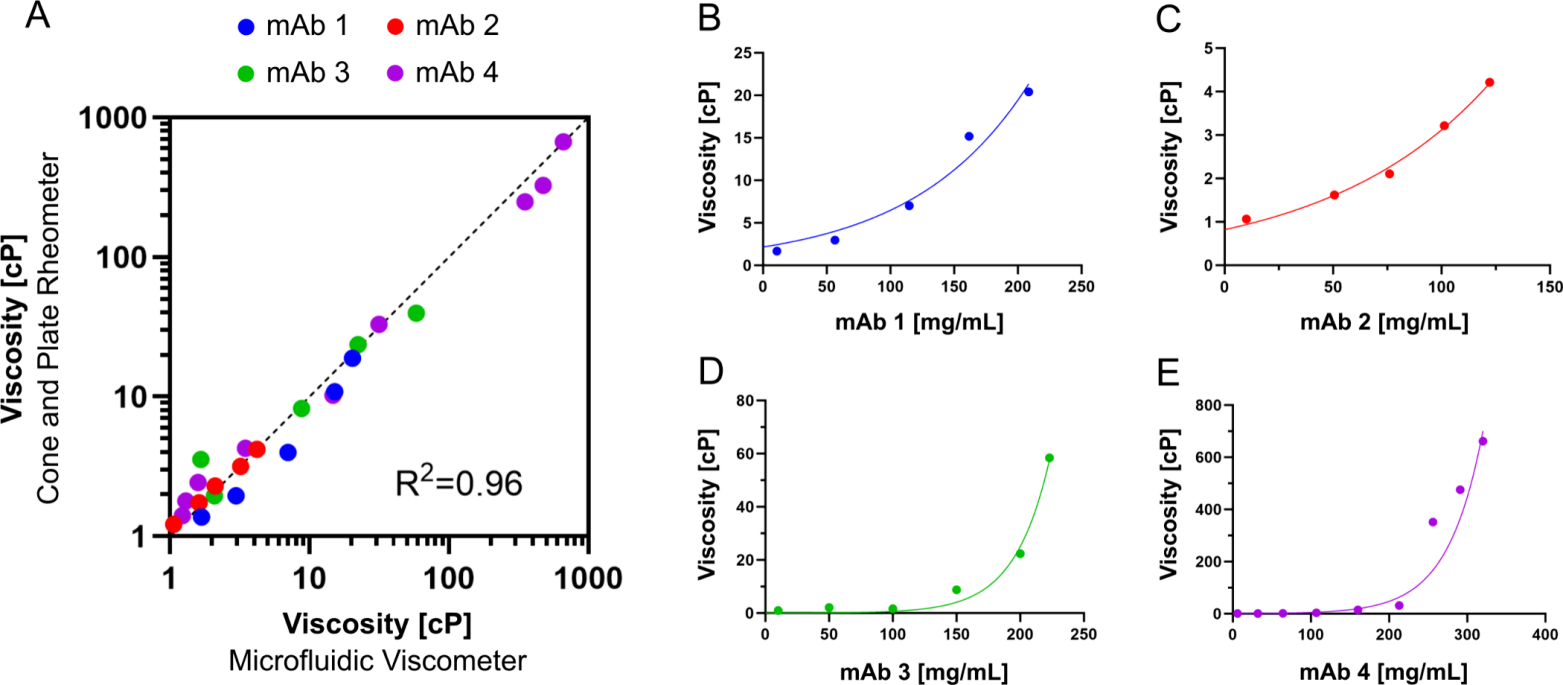
(A) Correlation between viscosity values measured using the microfluidic viscometer and the gold-standard rotational cone and plate rheometer. Measurements were performed as blind experiments. Different colors indicate the four different antibodies measured at various concentrations. (B–E) Viscosity measurement as a function of antibody concentration for the four analyzed antibodies (mAb 1 - mAb 4).

The viscosity of the four monoclonal antibodies is shown in Figure 4. In panel A, the viscosity measurements using the microfluidic viscometer are compared with the rotational cone and plate rheometer, and panel B-E the viscosity values for the different antibodies is shown as a function of antibody concentration. The data were fitted using an exponential function η = η_0_*e*^*kc*^, where η_0_ represents the viscosity of the formulation buffer without protein, *k* an exponential coefficient, and *c* the antibody concentration.

As can be seen in Figure 4A, the results demonstrate a remarkable agreement between the developed microfluidic platform and the gold standard cone and plate rheometer, with a correlation coefficient *R*^2^ = 0.96. Additionally, the high dynamic range—from 1 to over 600 cP—indicates that therapeutic proteins and antibody formulations can be measured using this technique.

### Discussion: Implications for Formulation Development and in Situ Quality Control

The developed method allows for the measurement of viscosity using very small sample volumes, which has significant implications for formulation development and devopability studies of therapeutic proteins. Notably, the developed viscometer reduces the required amount of antibody by a factor of 50 compared to gold-standard cone and plate rheometer measurements. This reduction not only decreases material consumption costs but also enables time savings by minimizing the preparation and handling steps involved with larger sample sizes. Importantly, our device eliminates the need for time-consuming cleaning procedures that are typically required by commercial rheometers. The single-use characteristics and parallelization offered by the microfluidic viscometer therefore accelerates the formulation development process by offering a high experimental throughput.

Furthermore, in contrast with current benchtop instrumentation, the developed microfluidic viscometer enables measurements at the point of sample collection through the combined use of a vacuum packaging method, a portable imaging device, and an automated image and data analysis for calculation of viscosity.

By offering a portable viscosity measurement, the developed viscometer can be implemented as a process optimization method, by enabling a rapid measurement and thus performing real-time adjustments in the manufacturing process. Moreover, the portable viscometer presents the opportunity to employ viscosity measurement as a in situ quality control tool, ensuring that the products meet the required standards before being used.

## Conclusion

In this work, we developed a single-use, low volume, and high throughput microfluidic-based viscometer allowing for fast, precise, and parallelized viscosity measurement up to 600 cP. We demonstrated the ability of rapid characterization of mAb viscosity using 10 μL sample volumes, showing excellent agreement with the gold-standard rheometer for viscosity measurement.

Given the absence of moving parts, the microfluidic viscometer is easy to manufacture and to use, and provides an accurate and rapid measurement of viscosity. This rapid screening ability enables the use of this platform in developability studies.

Furthermore, the implementation of vacuum-packing and smartphone-based image aquisition, combined with automated calculation of viscosity, ensures that the device can be used at the point of sample collection with limited equipment, opening applications for in situ quality control of therapeutic proteins, as well as viscosity measurement of human fluids for diagnostic applications.
